# Styrylpyrone Derivative (SPD) Extracted from *Goniothalamus umbrosus* Binds to Dengue Virus Serotype-2 Envelope Protein and Inhibits Early Stage of Virus Replication

**DOI:** 10.3390/molecules27144566

**Published:** 2022-07-18

**Authors:** Noor Zarina Abd Wahab, Nazlina Ibrahim

**Affiliations:** 1 School of Biomedicine, Faculty of Health Sciences, Universiti Sultan Zainal Abidin, Kuala Nerus 21300, Terengganu, Malaysia; 2 Department of Biological Sciences and Biotechnology, Faculty of Science and Technology, Universiti Kebangsaan Malaysia, Bangi 43600, Selangor, Malaysia; nazlina@ukm.edu.my

**Keywords:** anti-viral, DENV-2 E protein, *Goniothalamus umbrosus*, styrylpyrone derivative

## Abstract

A study was conducted to investigate the anti-viral effect of a styrylpyrone derivative (SPD) called goniothalamin and the effects on the dengue virus serotype 2 (DENV-2) replication cycle. The SPD was prepared from the root of *Goniothalamus umbrosus* after purification with petroleum ether. The isolated SPD was then subjected to gas chromatography–mass spectrometry (GC-MS) and nuclear magnetic resonance (NMR) analyses for structure validation. The cytotoxicity of the SPD was evaluated using a cell viability assay, while the anti-viral activity of the SPD towards DENV-2 was confirmed by conducting a foci reduction assay which involved virus yield reduction, time-of-addition, and time removal assays. Transcriptomic analysis via quantitative real-time polymerase chain reaction (qRT-PCR) using the DENV-2 E gene was conducted to investigate the level of gene transcript. Immunocytochemistry analysis was used to investigate the effects of SPD treatment on protein E expression. Finally, software molecular docking of the SPD and E protein was also performed. The cytotoxicity assay confirmed that the SPD was not toxic to Vero cells, even at the highest concentration tested. In the time-of-addition assay, more than 80% foci reduction was observed when SPDs were administered at 2 h post-infection (hpi), and the reduction percentage then dropped with the delay of the treatment time, suggesting the inhibition of the early replication cycle. However, the time removal assay showed that more than 80% reduction could only be observed after 96 h post-treatment with the SPD. Treatment with the SPD reduced the progeny infectivity when treated for 24 h and was dose-dependent. The result showed that transcript level of the E gene in infected cells treated with the SPD was reduced compared to infected cells without treatment. In immunocytochemistry analysis, the DENV-2 E protein exhibited similar expression trends, shown by the gene transcription level. Molecular docking showed that the SPD can interact with E protein through hydrogen bonds and other interactions. Overall, this study showed that SPDs have the potential to be anti-DENV-2 via a reduction in viral progeny infectivity and a reduction in the expression of the DENV-2 E gene and protein at different phases of viral replication. SPDs should be further researched to be developed into an effective anti-viral treatment, particularly for early-phase dengue viral infection.

## 1. Introduction

Dengue infection is caused by the dengue virus (DENV) which belongs to the genus Flavivirus in the family Flaviviridae. There are four serotypes of dengue virus: DENV-1, DENV-2, DENV-3, and DENV-4 [1]. Despite the variations, infection with each of the dengue serotypes results in the same disease and range of clinical symptoms. The genome contains positive-sense, single-stranded RNA molecules of approximately 11 kb in length which code for the viral proteins. Three are structural proteins: the capsid (C), envelope (E), and membrane (M) proteins. Seven are nonstructural proteins: NS1, NS2A, NS2B, NS3, NS4A, NS4B, and NS5. These nonstructural proteins play roles in viral replication and assembly [2]. DENV is transmitted principally by the *Aedes aegypti* mosquito. Dengue virus infection may be asymptomatic or may lead to undifferentiated fever, dengue fever (DF), or dengue hemorrhagic fever (DHF) with plasma leakage which may lead to hypovolemic shock (dengue shock syndrome, DSS) [3].

At present, no natural ingredients have been certified to treat dengue infection, although many traditional medicinal plants have been reported to have strong anti-viral activity, and some of them have already been used to treat animals and people who suffer from viral infection by inhibiting the replication of several viruses [4,5,6]. For example, *Carica papaya* or papaya has been used in traditional medicine as an alternative to treat dengue infection in humans [7]. Infection by DENV can result in thrombocytopenia which is characterized by a reduction in the number of blood platelets to less than 150,000 per µL of blood. This is due to bone marrow suppression, platelet destruction, platelet dysfunction, coagulation imbalance, and fibrinolysis [8,9]. In vivo studies have shown that an aqueous extract of *C. papaya* leaves was found to have an effect in increasing platelet count in study mice infected with DENV-2 [10]. In addition, flavonoids and other phenols found in *C. papaya* leaf extract can have a healing effect by compensating for mineral deficiency caused by DENV infection and strengthening the immune system against DENV infection [11,12]. *Euphorbia hirta* is another popular plant that is used in traditional medicine to treat dengue fever by people in the countryside areas of the Philippines [4]. A commercial capsule formulation has been developed for local Philippines communities’ usage in dengue infection treatment [13]. 

*Goniothalamus umbrosus*, locally known as `kenerak’ in Malaysia, is a tropical plant under the Annonaceae family which is well-distributed across Peninsular Malaysia. This plant has long been used traditionally as contraceptive, anti-pyretic, anti-malaria, and as post-partum treatment [14,15]. Water decoctions of dried flowers, leaves, and stem bark are usually used to treat ailments. Phytochemical constituents of *Goniothalamus* sp. have been studied quite extensively, resulting in the isolation of alkaloids, steroids, terpenoids, and cardiac glycosides from bark and leaf extracts of this plant. 

Phytochemical compounds have been recognized to have medicinal significances. For example, many alkaloids derived from medicinal plants show biological activities such as anti-viral, anti-bacterial, anti-inflammatory, and anti-cancer properties [16]. Steroids derived from plants are known to have anti-cancer [17], cytotoxic [18], immunosuppressive [19], and anti-microbial [20] properties. Tannins are known to have anti-viral properties against a large spectrum of viruses, anti-bacterial properties [21], and anti-tumor activity towards various tumors such as prostate and cervical cancers [22,23]. Cardiac glycosides have been used to treat congestive heart failure [24]. 

**Figure 1:**
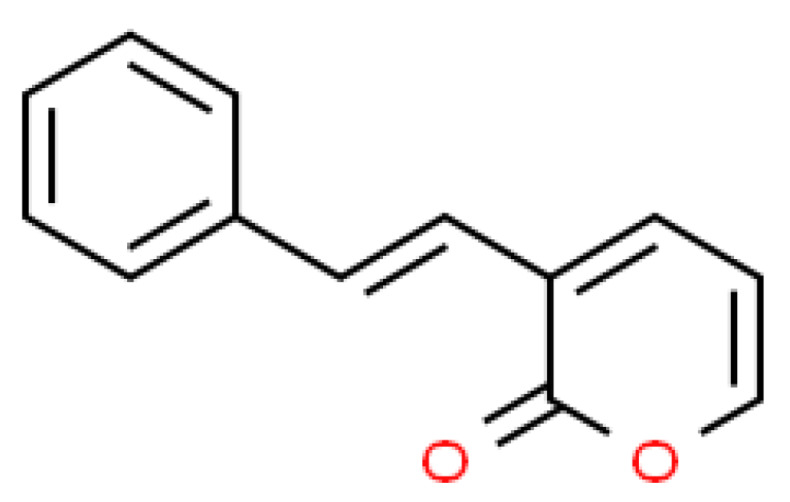
Basic structure of styrylpyrone.

A study investigated the chemical components in hexane and ethyl acetate extracts of *Goniothalamus* sp. and found a total of 110 compounds based on gas chromatography–mass spectrometry (GC-MS) detection [25]. These compounds include naphthalene derivatives, eudesma-4(14), 7(11)-diene, 1-butyl-2-cyclohexen-1-ol, benzaldehyde, and globulol. These compounds have been proposed to exert anti-bacterial and anti-cancer potentials. Another group of phytochemicals in *Goniothalamus* sp. is styrylpyrone derivatives (SPDs), one of which is goniothalamin. Styrylpyrone derivatives are among styryl lactones that have been isolated from several *Goniothalamus* sp. [26]. SPDs are secondary metabolites which fall under the styryl-lactones group in the polyketides class. The basic structure includes six-membered aromatic or 2-pyrone rings and oxygen function at the meta position [27]. This compound is distributed among plants in the Lauraceae, Annonaceae, and Piperaceae families [28] and also certain mushrooms [29]. Figure 1 shows the basic structure of styrylpyrone. This structure becomes the core skeleton for other types of styryl-lactones such as furano-pyrones and pyrano-pyrones [30]. This phytochemical compound has been reported to have potent anti-viral activity against herpes simplex virus type 1 [31,32], an anti-cancer effect, and neurotoxicity [33].

The identification of a pure compound can be determined using gas chromatography–mass spectrometry (GC-MS) and nuclear magnetic resonance (NMR). NMR spectroscopy is an analytical chemistry technique used to determine the structure, dynamics, reaction state, content, and purity of an organic compound [34]. GC-MS is a technique which involves compound separation in a gas chromatograph and the detection of fragmented molecules in a mass detector [35]. It is a separation technique of choice for smaller volatile and semi-volatile organic molecules such as alcohols, hydrocarbons, and aromatics, as well as steroids, fatty acids, pesticides, and hormones [36].

We have previously reported that SPD isolated from *G. umbrosus* was observed to be safe and non-toxic to cells, protect cells against viral infection, inhibit viral attachment and penetration, and also have a virucidal effect [37]. This study was conducted to further determine and elucidate the anti-viral effect of this active compound to the in vitro inhibition of different stages of the DENV-2 replication cycle and progeny infectivity. This is the first study to report that SPD has the potential to be anti-DENV-2 through the following modes: the interference of virus attachment and penetration into cells, direct destruction to the viral particle, the inhibition of viral progeny infectivity, and reducing the expression of DENV-2 genes and proteins at different phases of viral replication. 

## 2. Results

### 2.1. Isolation of SPD

Pure SPD (0.05 g) was isolated from *G. umbrosus* after purification with petroleum ether. The characteristics of an ununiformed clear crystal with pointed/sharp ends were observed. Tiny, needle, white and solid crystalline structure of SPD was obtained after several recrystallization using petroleum ether. This crystal was then subjected to GC-MS and NMR analysis for structure validation. The purity of the isolated SPD was validated using GC-MS. The GC-MS spectrum showed one peak with a mass to charge ratio (*m*/*z*) of 200 which was consistent with the SPD’s molecular weight [38]. Our result corresponded to molecular weight and fragmentation reported previously [39]. Major fragmentations are listed in Table 1. 

**Figure 2:**
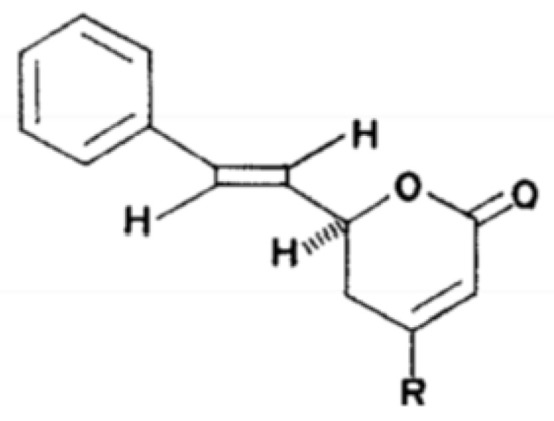
Goniothalamin structure.

The ^1^H NMR spectra for the isolated SPD in this study showed the signal at δ 7.0 for H-6 and δ 6.0 for H-3 as olefinic protons of lactone skeleton. Two more olefinic protons appeared at δ 6.4 and δ 6.7 which had H-4 and H-5 protons, respectively. The signal that appeared at δ 5.1 was an H-2 proton. The signal at δ 7.3 was a proton of the phenyl group with an H-8 proton. The structure of the SPD determined in this study was similar to the structure of goniothalamin (Figure 2) which was originally discovered [26]. 

### 2.2. Cytotoxicity of SPD

**Figure 3:**
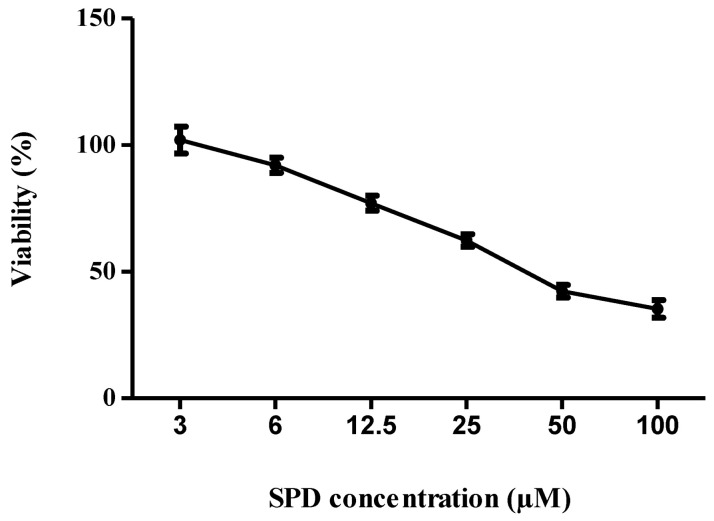
Cytotoxicity analysis of SPD. Graph shows percentages of cell viability at different SPD concentrations. Cytotoxicity concentration of SPD that killed 50% of Vero cells. Data represent mean values ± SEM (*n = 3*).

The cytotoxic concentration, CC_50_, of the SPD that killed 50% of the Vero cell population was determined from the graph of cell viability percentage against SPD concentration. The CC_50_ value of the SPD was 0.0085 mg/mL (42.5 µM), as shown in Figure 3. The graph shows the percentages of cell viability at different extract concentrations. 

### 2.3. Mode of Action of SPD

**Figure 4:**
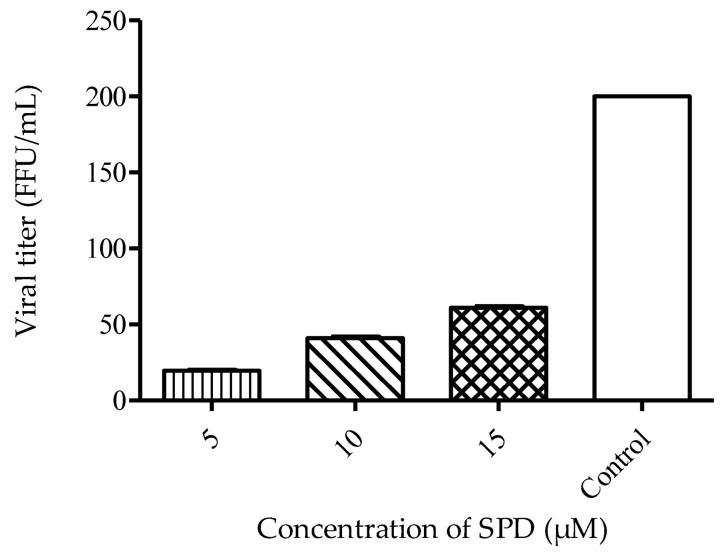
Viral yield reduction assay shows a dose-dependent pattern in foci inhibition. Data represent mean values ± SEM (*n* = 3).

The anti-viral study was conducted using focus reduction assay. The calculation of focus inhibition is the basis of calculating the percentage of virus reduction. Time-dependent studies were conducted to evaluate the effect of delayed treatment and also the effect of different time exposures to the SPD against its DENV-2 anti-viral activity. In the viral yield reduction assay, a concentration-dependent reduction in virus titer percentage was observed after 48 h post-treatment with SPD. The calculated virus titer revealed significant differences between all SPD concentrations. The treatment with the highest concentration (15 μM) resulted in the lowest titer produced, as shown in Figure 4. 

**Figure 5:**
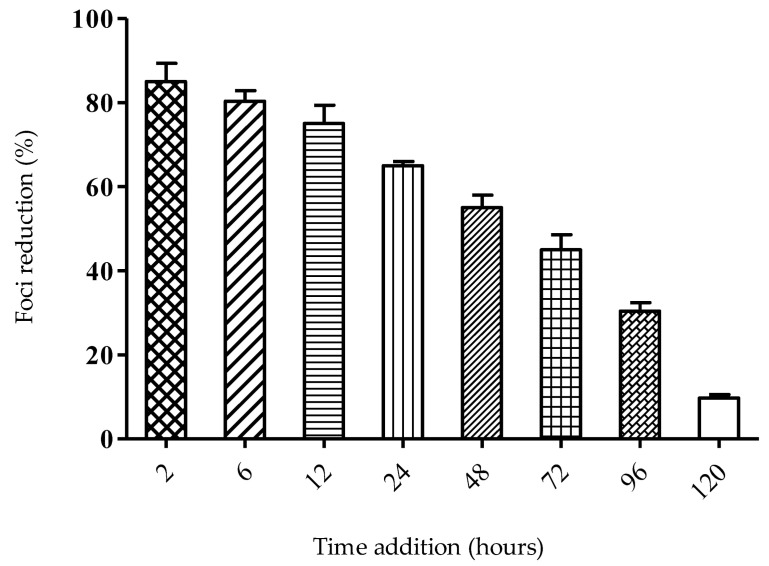
Time-of-addition assay. Graph shows activity of SPD when treatments were added at different times after infection. Data represent mean values ± SEM (*n* = 3).

In the time-of-addition study, SPD treatment was observed to be most effective when added at two hours post-infection (hpi) (Figure 5). More than 80% focus reduction was observed when the SPD was administered at 2 hpi, and the reduction percentage gradually dropped at later time points. Since the SPD’s optimum activity was when added at 2 hpi, the most probable target of SPD is in the inhibition of one of the stages in the viral early replication cycle.

**Figure 6:**
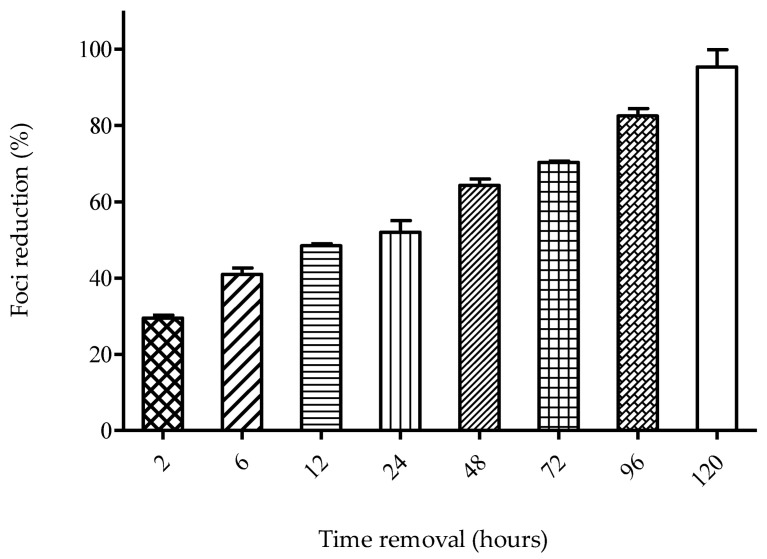
Time removal assay. Treatment was removed at designated time point to evaluate the effect of different time of exposures of SPD. Data represent mean values ± SEM (*n* = 3).

A time removal assay was carried out to evaluate the minimum time of SPD treatment that conferred the optimum anti-DENV-2 activity. Treatment with the SPD began at 2 hpi and the removal of the SPD continued for every three hours until 15 hpi. Low plaque reduction was observed as early as 6 hpi (four hours post-treatment) (Figure 6). As early as 9 hpi (seven hours post-treatment), 50% plaque reduction was observed, and plaque reduction reached its peak after exposure of 15 hpi.

### 2.4. SPD Alters E Gene during DENV-2 Replication

**Figure 7:**
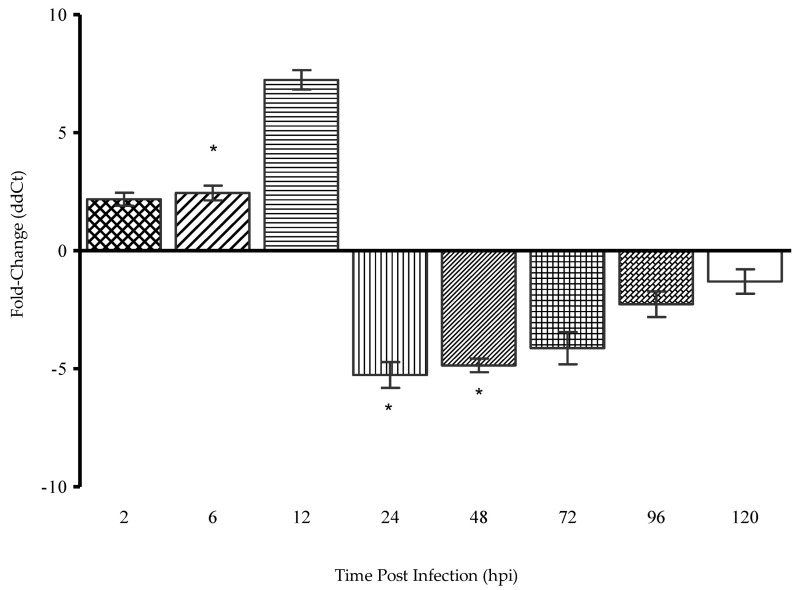
Calculated relative expression in SPD-treated virus relative to the expression of non-treated virus of E gene at 2, 6, 12, 24, 48, 72, 96, and 120 hpi. (*) indicates statistical significance min *p* value < 0.05. Data represent mean values ± SEM (*n* = 3).

Quantitative RT-PCR was employed to monitor the transcription levels of the DENV-2 E gene within 120 h of replication in Vero cells. The effect of the SPD treatment at the concentration of 12.5 µM on DENV-2 gene expression was evaluated from time interval samples of 2, 6, 12, 24, 48, 72, 96, and 120 hpi. The E gene was selected as the target gene in this study to represent the early phase in the DENV-2 replication cycle. The E gene is required in the process of attachment and entry of the virus into the host cell [40,41]. The assessment of gene expression levels was made relatively by comparing the cycle threshold (Ct) values of untreated infected cells with the Ct values of treated infected cells after normalization. Treated uninfected cells and uninfected as well as treated cells were the negative controls of the experiment, while cells infected with DENV-2 at 200 foci forming units (FFUs) were the positive controls of this experiment. Figure 7 shows the relative gene expression levels for the E gene based on eight different times. The results of the early phase of infection (2 to 12 hpi) showed that the expression of the E gene in treatment samples was inhibited. The rate of E gene expression was relatively low at 2 hpi, 1.0 ∆∆ct. The rate of E gene expression continued to decrease to 2.9 ∆∆ct compared to the untreated sample. However, after 24 hpi, the rate of inhibition of its expression decreased drastically. Decreased expression for the E gene at 2 hpi was insignificant with *p* value = 0.4392. However, the decrease in E gene expression was significant (*p* < 0.05) at 6 hpi. It is likely that the SPD needed time to be absorbed and act on the E gene.

### 2.5. SPD Inhibits DENV-2 E Protein

**Figure 8:**
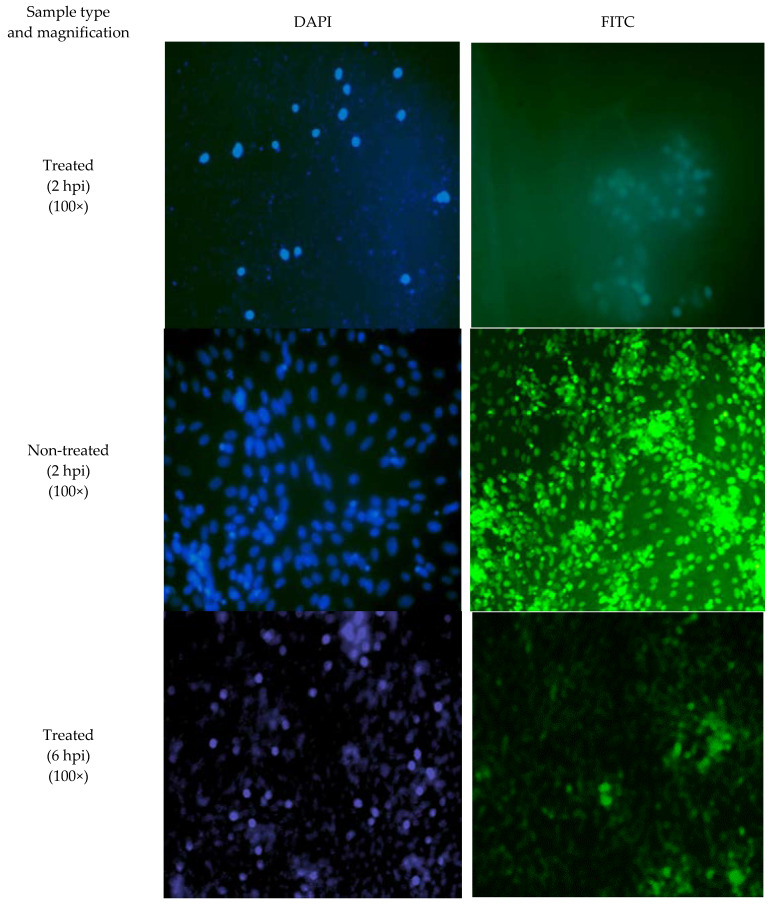

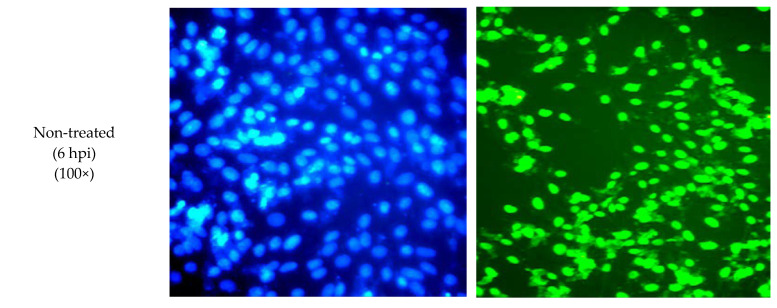
Representative images of SPD-treated and non-treated virus-infected cells’ immunofluorescence staining using DAPI and FITC conjugated to anti-E at 2 and 6 hpi.

Immunofluorescent staining analysis was performed to confirm the results of quantitative RT-PCR analysis and, at the same time, to prove the effect of the SPD on the attachment process involving the DENV-2 E protein in infected and treated cells. Figure 8 shows the observations of infected cells with and without the SPD treatment under a fluorescence microscope for cells stained with 4′, 6-diamidino-2-phenylindole (DAPI) (blue color) and labeled with antibody E (fluorescein isothiocyanate or FITC, green color) at 2 hpi. The number of cells and the intensity of blue and green colors were found to be reduced in infected cells treated with SPD compared to infected cells without treatment. At 6 hpi, significant differences could be observed in terms of cell number and blue and green color intensity for untreated cells and infected cells treated with SPD. These differences indicate SPD affects the accumulation of protein E at 2 and 6 hpi. This proves that the disruption of viral glycoproteins indirectly decreased the potential of the virus in infecting cells [42]. The results obtained from the immunofluorescent test on protein E confirm the results of the quantitative RT-PCR analysis that was performed previously.

### 2.6. SPD Binds to Domain III of DENV-2 E Protein

**Figure 9:**
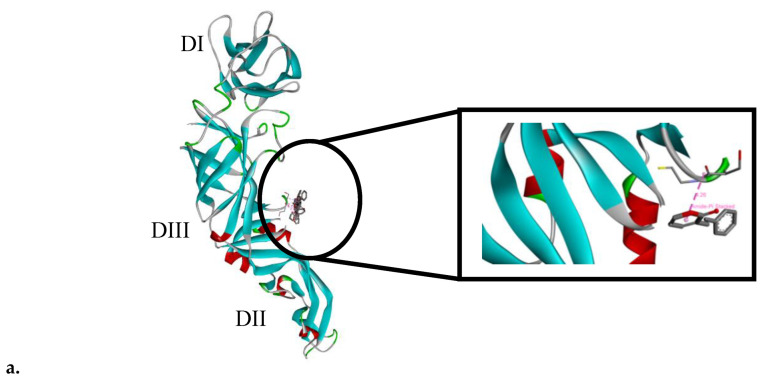

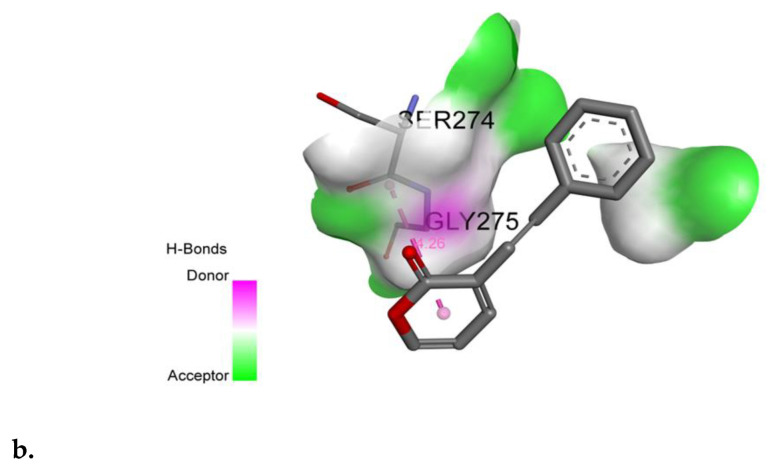
Molecular docking of SPD. (**a**) Binding pose of SPD molecular structure on DENV-2 E protein (PDB entry: 4u2c). Arrow indicates the molecular structure of SPD. Domain III is marked red. (**b**) Interaction of SPD with the amino acid residues of DENV-2 E protein as shown by docking. SPD binds at domain III of E protein which ranges from amino acid residues 274 to 275, by forming a hydrogen bond with the amino acid residues of the E protein.

The molecular docking of the SPD was carried out on the DENV-2 E protein. SPD was shown to bind to domain III of the DENV-2 E protein, spanning from amino acid residues 274 to 275, at a binding affinity of −1.86 Kcal/mol. The binding pose of the SPD on DENV-2 E protein is shown in Figure 9a, while its interaction with the amino acid residues of the protein is shown in Figure 9b. 

## 3. Discussion

Goniothalamin was first reported as an active constituent of the bark of G. *andersonii*, *G. macrophyllus* Miq., and *G. malayanus* [26]. Since then, the occurrence of goniothalamin has been confirmed in stem bark of *G. cardiopetalus* Hook.f. & Thoms [43], aerial parts of *G. amuyon* [44], and roots of *G. griffithii* [45]. Petroleum ether has been shown to be an effective solvent in the extraction of goniothalamin from *Goniothalamus* sp. This was proven when 5-isogoniothalamin oxide was successfully isolated from the stem bark of *G. sesquipedalis* [46]. Here, we evaluated the potency of the SPD or goniothalamin extracted from the root of *G. umbrosus* against DENV-2.

Pure compounds can be isolated from crude extract. Crude extract is usually prepared via several means, namely maceration, decoction, digestion, infusion, percolation, and hot continuous extraction (using Soxhlet apparatus). In this current study, we used hot continuous extraction using Soxhlet apparatus because this technique poses advantages due to its simple approach, low cost, and efficient extraction owing to the constant displacement of fresh solvent which comes in contact with plant material. The further isolation of pure SPD from crude extract was achieved by using simple crystallization from crude extract [47]. Petroleum ether was chosen as the solvent in this study because it is commonly used due to its relatively low cost compared to other organic solvents. Furthermore, it has a low boiling point. It is less hygroscopic than diethyl ether, less flammable than diethyl ether, and more selective for hydrophobic lipids than diethyl ether. Petroleum ether can also be used instead of heptane for fat extraction [48]. The results from GC-MS and NMR confirmed the crystal isolated from *G. umbrosus* as goniothalamin (SPD) based on a previous study [39] with a confirmed structure similar to previous results [26]. No obvious contamination peak was observed via the NMR spectrum with a 100% peak observed in mass-spectroscopy data at the *m*/*z* value of 200, which confirmed that the isolated compound was pure. The stability evaluation of the SPD was conducted based on the physical appearance, pH, and drug content according to a previous study [49].

A cytotoxicity assay was performed ahead of anti-viral screening to determine the SPD concentration that was cytotoxic to 50% of the cell population (CC_50_). The cytotoxicity analysis via the crystal violet dye assay was made concurrently with the anti-viral activity assessment to ensure that the potential virus-specific compound had low or no effect on cellular metabolism and thus showed no toxicity effect against the host cell [50]. From this CC_50_ value, lower concentrations which were least toxic or non-toxic to the cells were used in anti-viral screening. In this study, a 3-(4,5-dimethylthiazol-2-yl)-2,5-diphenyltetrazolium bromide (MTT) assay was utilized in the cell viability assessment by measuring the ability of mitochondria to reduce tetrazolium salt to insoluble formazan crystal [51]. The SPD’s CC_50_ was considered to be not in the range of cytotoxic compounds since the value of CC_50_ was determined to be higher than the minimum value for a compound with active cytotoxicity. Any CC_50_ or IC_50_ value of a compound that was less than 4 μg/mL was considered to have an active cytotoxic effect [52]. 

A virus yield reduction assay was used to determine the infectivity of the virion produced by initial infection. Titration was performed to investigate the reduction in virion produced after treatment with the SPD. This assay is a powerful method to validate potential anti-viral drugs by evaluating the ability of the progeny virus to re-infect cells after exposure to SPD treatment [53]. A yield reduction assay validated the anti-viral activity of the SPD with a reduction in virus progeny. The foci reduction was found to be dependent to the SPD concentration and virus dose. This is an expected and common scenario in most anti-viral studies where drug concentration and virus dose infection affect the competency of anti-viral activity [54,55,56]. All further testing on the SPD’s anti-viral activity was conducted using 12.5 μM as the anti-DENV-2 standard concentration. A similar SPD concentration (12.5 μM) was used previously [57], which shows the inhibition of HSV-1 at higher viral load infections.

Time-dependent studies were conducted to evaluate the effect of delayed treatment and also the effect of different time exposures to the SPD against DENV-2 anti-viral activity. Since the SPD’s optimum activity is when added at 2 hpi, the most probable target for the SPD is the inhibition of one of the stages in the early replication cycle of the virus. A time removal assay was conducted to evaluate the minimum time of SPD treatment that conferred the optimum anti-DENV-2 activity. The efficacy of the SPD as an anti-DENV-2 agent depends on the time of exposure. At a minimum, only two hours of treatment is enough to allow the SPD to prevent 29% of foci formation. This is likely due to effective concentrations of SPD being able to be absorbed easily and quickly by the cells to prevent the formation of focus and thus prevent viral replication from occurring. The time-of-addition assay showed that the SPD inhibited viral replication up to 120 h after infection. It is suggested that the SPD is effective when added at the beginning as well as at the end of an infection. This may be due to the inhibition of the viral envelope process at the beginning of infection and the disruption of viral replication at the end of infection. Referring to the results of the time removal assay, the percentage of focus formation reduction increased in the duration between 2 to 120 h. The reduction in focus formation could be seen as early as 2 hpi. and continued to increase in proportion to the time of exposure to the treatment given. The longer the SPD is exposed to DENV-2-infected cells, the higher the rate of foci formation reduction. 

The potential of the SPD as a good anti-viral agent against DENV-2 could be determined by looking at its different mechanisms for stopping viral infections. The E gene was selected in this study because the ability of an anti-viral agent to target receptors on host cells or on attachment factors can inhibit the adhesion and adsorption of viral proteins to the host cells, thereby preventing viral fusion and entry into host cells that ultimately stop DENV entry into host cells. Moreover, the E gene encodes the proteins necessary in the early phases of infection that play a role in the regulation of viral adhesion and entry into host cells. The decreased expression of the E gene transcript can be observed in qRT-PCR results starting from 2 to 6 hpi. These results are in line with observations made in the time-of-addition assay which showed the most significant reduction in focus formation is at the initial infection. The time-of-addition assay that was conducted explains the stage at which the SPD affects the DENV-2 replication cycle. A time-of-addition test determines how long the addition of a test substance can be delayed before it loses its anti-viral activity [58]. The immunostaining of infected cells confirmed that the SPD inhibited the DENV-2 E protein. Reduced E protein expression was observed when treated with the SPD compared to non-treated samples. The immunofluorescent staining confirms that SPD treatment influences E protein expressions. Staining with DAPI indicated that nuclear staining for treated and infected cells was reduced compared to non-treated and infected cells.

The cycle of viral infection begins with the interaction of viral structural proteins, mediated by the E proteins that bind to the host cell receptors or attach to adhesion factors to facilitate viral entry. Glycoprotein E in DENV is the most important molecule during the viral entry process because it is responsible for identifying and binding host cells, clathrin-intermediate endocytosis, and subsequently fusing the viral membrane and host cells. These proteins are able to interact with various cell molecules. Therefore, binding is considered an ideal target for the development of anti-virus agents [59,60]. Several parts of the DENV’s E glycoprotein may be suitable as drug targets, including the stem region, the hydrophobic pocket, and the receptor III binding domain. Restrictions on any particular part can interfere with the process of DENV entry and adsorption to the host cells. The inhibitory reaction that occurs early in the cycle of infection will interfere the process of infection, maturation, and spread of the virus. Molecular docking analysis showed that the SPD is a potential inhibitor of the DENV-2 protein. This agrees with the results obtained in in vitro anti-viral tests and gene and protein expression analyses that were conducted. In silico molecular docking analysis showed that the SPD inhibited the activity of protein E in the DENV-2 replication cycle. Molecular docking analysis is a good approach to predict and match targeted ligands and proteins to understand the possible conformational changes and subsequently explain the overall interactions involved [61]. The SPD was found to inhibit the DENV-2 replication cycle through its inhibitory effect on the E gene and protein. This study provided further understanding of the interaction of the SPD as an inhibitor to the DENV-2 E protein which are beneficial in the development of anti-DENV agents.

## 4. Materials and Methods

### 4.1. Synthesis of SPD

5-hydroxy-7-phenylhepta-2,6-dienoic acid-δ-lactone (SPD) was isolated from *Goniothalamus umbrosus*. In brief, 300 g of dried root of *G. umbrosus* was extracted in petroleum ether using Soxhlet continuously for seven days or until the solvent became colorless. Crude extract was concentrated with rotary evaporator (Laborata 4000, Heidolph, Germany) and was further purified with petroleum ether to yield a crystal compound of the SPD. The purity of the compound was confirmed via FT-NMR 400 MHz (Bruker Avance 111) and gas chromatography (QP2010 Ultra) using the SPD from [57] as the standard. 

### 4.2. Cytotoxicity Assay

The assay was performed as described in a published study [62]. The cytotoxicity assay was conducted on monolayer Vero cells grown in a 96-well plate utilizing 3-(4,5-dimethylthiazol-2-yl)-2,5-diphenyltetrazolium bromide (MTT) for colorimetric analysis [63]. Vero cells were seeded at 2 × 10^4^ cells per well in 96-well, flat-bottomed microtiter plates (SPL Life Sciences, Pocheon-si, Korea) supplemented with 5% fetal bovine serum (FBS) and incubated at 37 °C overnight. Upon confluency, growth medium was removed and 100 μL of SPD from several concentrations (3–100 µM) was added. Cells were further incubated at 37 °C in a 5% CO_2_ atmosphere for 48 h. Cells without compound treatment served as base controls. After 48 h of incubation, the cells were washed gently, stained with MTT (5 mg/mL) for a minimum of two hours, and lysed with dimethyl sulfoxide (DMSO) to dissolve the formazan crystals. Color development was detected using a microplate reader (TECAN Infinite 200 PRO) at 540 nm. The percentage of living cells was calculated via comparison with healthy untreated cells. A dose–response curve was plotted using Graph Pad Prism 5, and the half maximal cytotoxic concentration (CC_50_) of the SPD was determined from the plot. The concentration of the SPD which showed at least 80% cells’ viability in the cytotoxicity assay was chosen as the starting concentration used throughout the experiments. This concentration was important for the anti-viral assays in the next step. 

### 4.3. Anti-viral Mechanism Assay

The anti-viral evaluation of the SPD towards DENV-2 was conducted using a standard foci reduction assay based on a published study [64] with slight modification. The evaluation was divided into an anti-viral screening and mechanism study. Anti-viral screening was composed of a post-treatment study, pre-treatment study, and virucidal study (data from this previous study have been published), whereas the anti-viral mechanism was composed of a virus yield study, time-of-addition study, and time-of-removal study. The concentration of the SPD used in this current anti-viral mechanism assay was based on the anti-viral screening result obtained in the previous study [37]. 

#### 4.3.1. Virus Infectivity Assay

The assay was conducted according to a published study [65] with modifications. Cells were infected with DENV-2 at 200 FFU for two hours at 37 °C. Infected cells were treated with different concentrations of the SPD (5, 10, and 15 µM) and further incubated at 37 °C for ten hours. At the end of the incubation, infected cells were frozen at −80 °C and thawed for five minutes repeatedly three times. This was to ensure that the virus in the infected cells stopped replicating. Cells were detached from wells via sonication for 15 min, and the virus was collected via centrifugation in the supernatant. Foci were counted, and the titer reduction percentage was calculated by subtracting the titer of negative control from the test titer divided by the titer of negative control. The number of DENV-2 foci was counted using a stereomicroscope, and the titer of virus was expressed as foci forming units (FFUs). The percentage of foci inhibition was calculated using the following formula:

RF(%) = (C−T) × 100/C

where C is the mean of the number of foci for the negative control well (without compound), and T is the mean of the number of foci in the treated wells.

#### 4.3.2. Time-of-Addition Assay

Antiviral activity was determined by measuring the reduction in the number of viral foci by following a previously described assay [66]. Monolayer cells were infected with 200 FFU of virus and incubated at 37 °C for 2 h. The SPD (12.5 µM) was added at 2, 6, 12, 24, 48, 72, 96, and 120 h post-infection (hpi) followed by the addition of 1% methyl cellulose (MCS). Cells were further incubated at 37 °C until 24 hpi. Foci were stained with crystal violet solution (0.4%, *w*/*v*). The number of DENV-2 foci was counted using a stereomicroscope, and the titer of virus was expressed as foci forming units (FFUs) [67]. The percentage of foci inhibition by the virus released by the cells was titrated and calculated as discussed above.

#### 4.3.3. Time-Removal Assay

The assay was conducted according to a previous study [68] with modifications. Confluent monolayers of Vero cells were infected with 200 FFU of DENV-2 for two hours at 37 °C. The SPD (12.5 µM) was added to the infected cells immediately after treatment. A medium containing the SPD was removed at 2, 6, 12, 24, 48, 72, 96, and 120 h post-infection (hpi), and cells were washed twice with DMEM without FBS for one minute and replaced with 1% MCS. Cells were further incubated for 48 h before staining with crystal violet solution (0.4%). The percentage of foci inhibition was calculated using the formula as discussed above. 

### 4.4. Quantitative Real-Time–Polymerase Chain Reaction (qRT-PCR)

This assay was conducted according to a published procedure [69], with minor modifications. Quantitative RT-PCR was performed to determine the effect of the SPD on the transcription of the selected DENV-2 E gene at different replication stages. Confluent cells were infected with DENV-2 at 200 FFU, treated with 12.5 µM SPD, and the infected cells were removed at 2, 6, 12, 24, 48, 72, 96, and 120 hpi. Viral RNA was extracted using a viral RNA extraction kit (Rneasy Plus Mini Kit, Qiagen). For quantitative RT-PCR (qRT-PCR), a one-step protocol was employed and performed on Applied Biosystems, Thermo Fisher, USA. Primer pairs were designed from the conserved region of the DENV-2 E gene: forward 5′-GGCCTCGACTTCAATGAGATGG-3′, reverse 5′-CCTGTTTCTTCGCATGGGGAT-3′. 

### 4.5. Molecular Docking

The docking of the SPD to the DENV-2 E protein was conducted using the program AutoDock Vina [70]. The three-dimensional structure of the DENV-2 E protein was retrieved from the Protein Data Bank (PDB entry: 4u2c), while the structure of the SPD was retrieved from the ChemSpider database (http://www.chemspider.com) (accessed on 25 July 2019).

### 4.6. Immunofluorescent Staining

The assay was conducted according to a published procedure [71] with slight modifications. Confluent cells were seeded on confocal slits and virus-infected at 200 FFU. SPD (12.5 µM) was added and incubated at 4 °C for 90 min. After incubation, cells were washed with phosphate-buffered saline (PBS), followed by fixation in 4% paraformaldehyde for 30 min at room temperature (RT) and incubation with methanol at 20 °C for 10 minutes. Cells were washed three times with PBS, followed by incubation with PBS-Triton X–FBS (PBTF) for ten minutes at RT. Cells were washed once with PBTF and blocked with FBS 10% for 90 to 120 min. Virus E glycoprotein staining was conducted by adding anti-E primary antibody in PBTF for four hours at RT. This was then followed by the addition of secondary antibody conjugated with fluorescein isothiocyanate (FITC). The nucleus was stained with 4′, 6-diamidino-2-phenylindole (DAPI). Images were viewed at three random fields in two technical replicates for each treatment using a fluorescence microscope (Olympus).

### 4.7. Statistical Analysis

Statistical analysis was performed using Graph Pad Prism for Windows, version 5 (Graph Pad Software Inc., San Diego, CA, USA 2005). Data were analyzed using the Student’s t-test. All analyses used a 95% CI (confidence interval), and the difference obtained was significant if the *p* value < 0.05.

## 5. Conclusions

In this study, the SPD was confirmed to have anti-DENV-2 activity when administered to DENV-2-infected Vero cells by inhibiting the mechanism of viral attachment. SPD is proposed to inhibit the E protein during the early stages of the virus life cycle and hence suppresses early viral gene transcription, replication, and translation. An in vivo experiment is recommended to be performed to determine the efficacy of the SPD for its therapeutic ability as an anti-dengue agent.

## Figures and Tables

**Table 1:** Fragmentation of SPD in GC-MS analysis.

Isolated SPD	Fasihuddin et al. [10]
200	200
172	172
131	131
115	115
104	104
91	91
63	68

## Data Availability

The data presented in this study are not available on request from the corresponding author.
